# Parliament and Publications

**Published:** 1911-02-11

**Authors:** 


### Parliament and Publications.

### OPENING OF PARLIAMENT.

Parliament was opened by the King in person on February 6. His Majesty's Speech forecasted legislation dealing with the relations between the two Houses of Parliament, the removal of the pauper disqualification for Old Age Pensions, and a provision for the insurance of the industrial population against sickness and invalidity, and for the insurance against unemployment of those engaged in trades specially liable to it. It is understood that the main principle of the sickness and invalidity scheme will be that of compulsory insurance for sickness and invalidity for the whole of the working population of the country whose incomes are below £160. The provisional scheme does not include medical or life insurance. Among the questions of which notice has been given is one by Mr. Goldman, who intends to ask the Secretary of State for War whether the attention of the War Office has been drawn to the cures effected by the use of Professor Ehrlich's preparation "606"; and whether any steps have been taken to employ this preparation in suitable cases in the British Army at home and abroad.

